# A comparative study of five physiological key parameters between four different human trophoblast-derived cell lines

**DOI:** 10.1038/s41598-017-06364-z

**Published:** 2017-07-19

**Authors:** Mario Rothbauer, Nilaykumar Patel, Hajnalka Gondola, Monika Siwetz, Berthold Huppertz, Peter Ertl

**Affiliations:** 1 Vienna University of Technology, Faculty of Technical Chemistry, Institute of Applied Synthetic Chemistry & Institute of Chemical Technologies and Analytics, Getreidemarkt 9, 1060 Vienna, Austria; 2 Medical University of Graz, Institute of Cell Biology, Histology and Embryology, Harrachgasse 21/VII, 8010 Graz, Austria; 3 University of Vienna, Department of Pharmacognosy, Althanstrasse 14, 1090 Vienna, Austria

## Abstract

The human placenta plays a crucial role as the interface between mother and fetus. It represents a unique tissue that undergoes morphological as well as functional changes on the cellular and tissue level throughout pregnancy. To better understand how the placenta works, a variety of techniques has been developed to re-create this complex physiological barrier *in vitro*. However, due to the low availability of freshly isolated primary cells, choriocarcinoma cell lines remain the usual suspects as *in vitro* models for placental research. Here, we present a comparative study on the functional aspects of the choriocarcinoma cell lines BeWo, JAR and Jeg-3, as well as the first trimester trophoblast cell line ACH-3P as placental *in vitro* barrier models for endocrine and transport studies. Functional assays including tight junction immunostaining, sodium fluorescein retardation, trans epithelial resistance, glucose transport, hormone secretion as well as size-dependent polystyrene nanoparticle transport were performed using the four cell types to evaluate key functional parameters of each cell line to act a relevant *in vitro* placental barrier model.

## Introduction

The human placenta plays a crucial role in the development and health of the fetus as the interface between mother and fetus by regulating nutrient and oxygen transport from the mother to the fetus as well as releasing fetal waste products into the maternal circulation. As hormone producing organ the placenta maintains and supports pregnancy^1^; however, due to its high metabolic activity biotransformation of xenobiotics to potentially toxic compounds may occur^2, 3^. The placenta is a very complex and unique organ that undergoes morphological as well as functional changes on the cellular and tissue level throughout gestation^4–6^. Even though this organ represents a rather weak barrier compared to other physiological barriers like the blood-brain-barrier^7^ or the Sertoli cell barrier^8^, it still can exhibit a protective function by preventing the interaction of potentially toxic entities including bacteria, viruses and parasites with the fetus^9–11^. Placental transport takes place at a multi-cellular interface between the mother and the fetus and mainly comprises specific components of the fetal villous tree: villous syncytiotrophoblast, villous cytotrophoblasts as well as placental endothelial cells, often referred to as the ‘placental barrier’^12^.

Human placental villi of the villous trees are covered by a highly specialized two-layered epithelium-like layer, the villous trophoblast. Special to the outer layer of villous trophoblast, also called syncytiotrophoblast, is that it differs from conventional stratified epithelia by absence of lateral cell borders and thus represents a real syncytium. The subjacent layer consists of mononucleated cells termed villous cytotrophoblasts. In the cytotrophoblast, proliferation is restricted to a small subset of progenitor cells, which divide asymmetrically and undergo differentiation resulting in fusion with the overlying syncytium^13, 14^. The conversion from the mononucleated to the syncytial state results in alterations of the trophoblast phenotype over time, resulting in the production of placental hormones such as human chorionic gonadotropin (hCG)^15^, human placental lactogen^4^, pregnancy-specific glycoprotein^16^ and leptin^17^. In the first trimester the cytotrophoblast cell population makes up a complete cell layer underneath the syncytium, however, starting mid gestation a reduction of this layer becomes obvious resulting in the formation of a mostly one layered villous trophoblast at the end of gestation. Hence, there is a continuous change from a double to a single layered villous trophoblast during pregnancy, changing the characteristics of this unique placental barrier^6, 18^.

So far, our knowledge of placental structure and function during pregnancy is limited and deeper understanding of the evolution and functionality of the human placenta throughout gestation is key to gain understanding on parameters that trigger placental dysfunctions. To better understand how the placental barrier works, to date, a set of *in vivo and in vitro* models for human placental transfer has been established including animal models^19^, *ex vivo* human placenta perfusion^20^, explant cultures^21^ as well as *in vitro* cell cultures^22–24^. The biggest issue with animal models is that the placental organ architecture has large interspecies variability and except for humans only primates, rabbits and rodents share the discoid type, while there are still major morphological, physiological as well as genetic differences^19, 25^. Although animal models closest to human include non-human primates such as chimpanzees and gorillas, use of these animals is highly questionable due to their endangered status. Therefore, most information on placental biology has to date been deduced from human placental tissue after delivery, pathological pregnancies and *ex vivo* model systems. For instance, the *ex vivo* human placental perfusion model, which was originally developed by Panigel and co-workers in 1962^26, 27^, provides a controlled system for studying trans-placental transport and is commonly used for pharmacokinetic studies. Although this model allows the investigation of molecule and material transfer on a whole-organ scale with organized tissue architecture^28–31^, it exhibits poor reproducibility, low throughput capabilities and no standardization at all. Furthermore, placental perfusions are normally carried out in a narrow time window of 2 to 6 hours since whole placental tissue viability is limited to a maximum of 24 hours^32^. Moreover, these *ex vivo* models are limited for placental studies of the third trimester of gestation because mostly delivered placentas are used for this experimental setup without the possibility to deduct transport mechanisms for the first and second trimester.

As mentioned earlier, the placenta is an organ that undergoes severe morphological changes during gestation that alter materno-fetal transport mechanisms severely^33^. To overcome these limitations, a lot of research effort so far has focused on how to actually recreate physiologically meaningful placental models using *in vitro* cell cultures. Even though these models have been successfully used to improve and understand placental metabolism and transfer^34–38^, they often fail to fully recapitulate the physiological architecture and microenvironment that influences transport processes^34, 35^. To overcome some of the shortcomings more delicate approaches including multi-cell cultures^39, 40^ and spheroid cultures^39, 41, 42^ have been developed. However, to reduce the use of or even replace animal models, today’s *in vitro* cell cultures need to improve with respect to organ function and physiological relevance.

As technology advances rapidly, more recently even three-dimensional bio-printed placental models^43^ and microfluidic placentas-on-a-chip^44, 45^ have emerged to overcome the limitations of conventional *in vitro* techniques and re-create a more relevant and physiological cellular microenvironment. For example, Miura and co-workers have demonstrated recently that fluid flow and thus shear stress has a tremendous impact on formation of placental microvilli^46^.

Even though a broad toolkit of techniques has been developed so far, it is evident that due to the low availability of fresh tissue samples and hence primary cell cultures, choriocarcinoma cell lines still remain a reasonable *in vitro* model for placental research since they are easy to handle and propagate. Even though these cells represent a cancer cell model, they still display various key capabilities of human placental trophoblast including hormone release^38, 47^, expression of glucose transporters^48, 49^ (e.g. GLUT-1, GLUT-3) as well as barrier capacity^50, 51^. However, only a handful of non-primary cell lines are available including several clones of choriocarcinoma cell lines such as BeWo^24^, JAR^38^, and Jeg-3^52^, as well as the more recently established human trophoblast cell line ACH-3P^23, 53, 54^. In this study, we present a comparative study on barrier and endocrine function of the above four different, well-established placental cell models.

## Results

### Placental barrier integrity is highly dependent on the placental cell model applied

**Figure 1:**
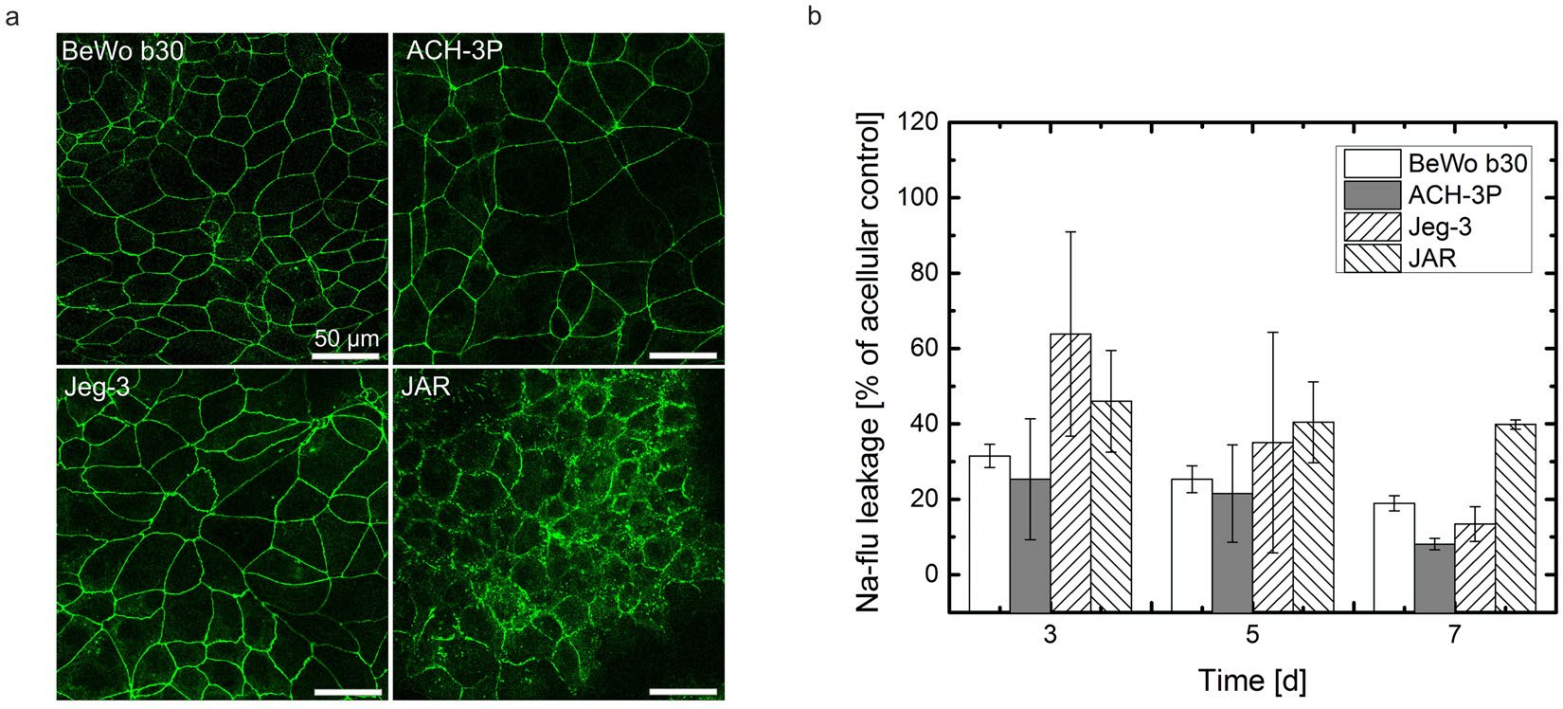
Barrier integrity of trophoblast cell lines. (**a**) Confocal laser scanning micrographs of tight junctions of BeWo, ACH-3P, Jeg-3 and JAR placental cell lines grown on gelatin-coated cover slides day 3 post seeding. Tight junctions are visualized using anti ZO-1 immunohistochemistry, scale bar is 50 µm. (**b**) Leakage of sodium fluorescein through BeWo, ACH-3P, Jeg-3 and JAR placental cell barriers on day 3, 5 and 7 post seeding. Data points are presented as mean values ± SD for *n* = *3* and expressed as % of acellular controls.

To assess the integrity of BeWo, ACH-3P, Jeg-3 and JAR cell barriers, formation of tight junctions and sodium fluorescein retardation/leakage were investigated for the different cell types. Results of immune fluorescence staining of ZO-1 (zona occludens-1) proteins are shown in Fig. 1a indicating that the localization of ZO-1 proteins is highly dependent on the cell type and day of culture. The cell lines BeWo, ACH-3P and Jeg-3 revealed properly formed tight junctions with sharp cell-to-cell boundaries, whereas JAR cells displayed heterogeneous and delocalized tight junctions at the intercellular boundaries 5 days post seeding on gelatin-coated cover slides. Surprisingly, all cell lines showed proper tight junction morphology at day 7 post seeding of trans well inserts (Supplementary Fig. 1).

To assess whether tight junction morphology directly affects barrier integrity, the apical-to-basal leakage of sodium fluorescein was evaluated at different time points using a transwell microtiter plate setup (Fig. 1b). For BeWo cells, sodium fluorescein transport was reduced to 31.49 ± 3.07%, 25.29 ± 3.55% and 18.90 ± 2.01% at day 3, 5 and 7 post seeding indicating a tight cellular boundary even at early stages of this *in vitro* barrier model. A tighter cell barrier was observed for ACH-3P cells with reduced transport to 25.29 ± 16.03%, 21.53 ± 12.95% and 8.12 ± 1.53%, respectively. Even though Jeg-3 cells displayed proper tight junction morphology, leakage of sodium fluorescein was elevated to 63.87 ± 27.09% and 35.04 ± 29.26% at day 3 and 5 post seeding. However, at day 7 post seeding sodium fluorescein leakage was reduced to 13.42 ± 4.60% indicating that the formation of a tight barrier is delayed for this cell type. However, sodium fluorescein leakage through JAR cell barriers decreased merely over time from 46.01 ± 13.47% at day 3 and 40.44 ± 10.7% at day 5 to 39.85 ± 1.22% at day 7.

**Figure 2:**
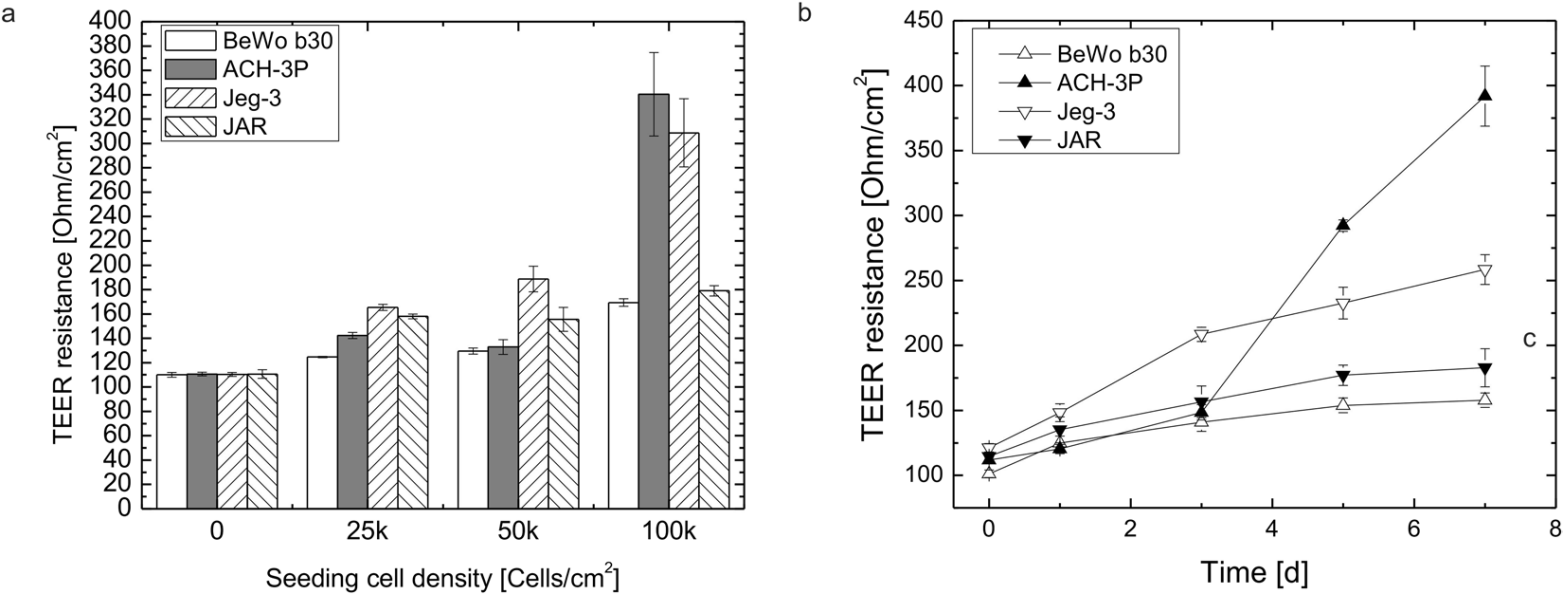
Barrier integrity of trophoblast cell lines based on trans-epithelial electrical resistance (TEER). (**a**) Influence of initial seeding density of BeWo b30, ACH-3P, Jeg-3 and JAR placental cells on electrical resistance on day 5 post-seeding. (**b**) Increase of electrical resistance of BeWo b30, ACH-3P, Jeg-3 and JAR placental cell barriers on day 1, 3, 5 and 7 post-seeding. Data points are presented as mean values ± SD for *n* = *6* and expressed as absolute values. Values on day 0 correspond to blank membrane values of acellular controls.

To further evaluate barrier integrity, trans-epithelial resistance measurements were performed every second day over a week in trans-well inserts also with varying initial cell seeding density (Fig. 2a). For formation of tight barriers, cell density during seeding has severe impact on the establishment of electric resistance values. BeWo b30 and JAR cells displayed a slight increase in TEER due to the increase in cell numbers with 169.33 ± 3.0 Ohms/cm^2^ and 179 ± 4.3 Ohms/cm^2^ at day 5 post-seeding, respectively. In contrast, increase of cell seeding density to 1 * 10^5^ cells per cm^2^ led to a further increase of electric resistance for ACH-3P and Jeg-3 cells with TEER of 340.4 ± 34.3 Ohms/cm^2^ and 308.6 ± 27.9 Ohms/cm^2^, respectively. To analyze the establishment of electric resistance over time in more detail, TEER was measured every second day for all cell lines at the highest seeding density with the highest TEER values. ACH-3P cells showed the tightest barrier formation with a 3.2-fold increase in resistance values with 120 ± 3.6 Ohms/cm^2^ at day 1 and 392 ± 23.0 Ohms/cm^2^ at day 7 post-seeding. Jeg-3 cells showed mediocre barrier formation with a 2.1-fold increase in resistance values with 121 ± 3.4 Ohms/cm^2^ at day 1 and 258 ± 11.5 Ohms/cm^2^ at day 7 post-seeding. JAR cells showed less barrier formation with a 1.3-fold increase in resistance values with 135 ± 9.9 Ohms/cm^2^ at day 1 and 182 ± 14.6 Ohms/cm^2^ at day 7 post-seeding. The slowest increase of barrier resistance increase showed BeWo b30 with a 1.2-fold increase in resistance values with 124 ± 5.3 Ohms/cm^2^ at day 1 and 157 ± 5.6 Ohms/cm^2^ at day 7 post-seeding. Even though the combination of time-resolved TEER measurements with the Na-Flu leakage assay is considered trustworthy for evaluation of cellular barriers, our results indicate that between the four different placental cell types there is no clear correlation between high TEER values and low Na-Flu values during the early stages of barrier establishment. However, on day 5 and 7 post-seeding Na-Flu retardation correlated very well with TEER values with highest integrity and tightness for ACH-3P and Jeg-3, followed by the lowest barrier integrity and tightness for BeWo b30 and JAR cells even though all cell types displayed proper ZO-1 immunofluorescence staining (Supplementary Fig. 1) with no difference in tight junction morphology at day 7 post TEER measurements.

### Glucose transport across placental cell barriers

**Figure 3:**
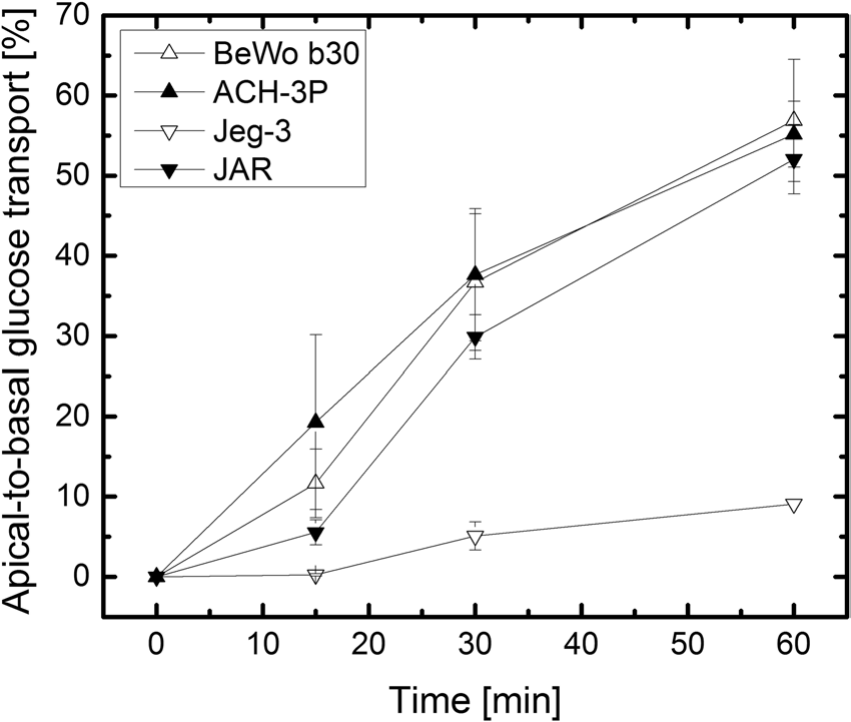
Influence of cell line type on transcellular transport of glucose. Apical-to-basal transport of 25 mM glucose through BeWo, ACH-3P, Jeg-3 and JAR cell barriers at day 5 post-seeding in transwell culture. Data points are presented as mean values ± SD for *n* = *3* and expressed as % of acellular controls (bare membrane inserts).

To evaluate the apical-to-basal transport of glucose through placental *in vitro* barrier models, glucose concentrations were analyzed for each cell type during 60 min of transport at day 5 post seeding in a transwell microtiter format and normalized to transport across acellular permeable transwell membrane inserts (Fig. 3). For acellular controls, glucose was observed to pass unhindered through the 3 µm membrane insert with transfer rates of 98.5 ± 1.5% at 15 min, 98.8 ± 0.7% at 30 min and 100 ± 0.2% at 60 min. BeWo and ACH-3P cells yielded steadily increasing glucose transfer rates peaking at 56.9 ± 7.6% and 55.2 ± 4.1% after 60 min, respectively. Glucose transfer was slightly reduced for JAR cells in comparison to BeWo and ACH-3P cells peaking at 52.0 ± 4.3% after 60 min. In contrast, Jeg-3 cells displayed the lowest capacity to transport glucose over the cellular barrier over 60 min with peak values of 9.1% ± 0.1%.

### Secretion of human chorionic gonadotropin and sensitivity towards chemical stimulation using forskolin

**Figure 4:**
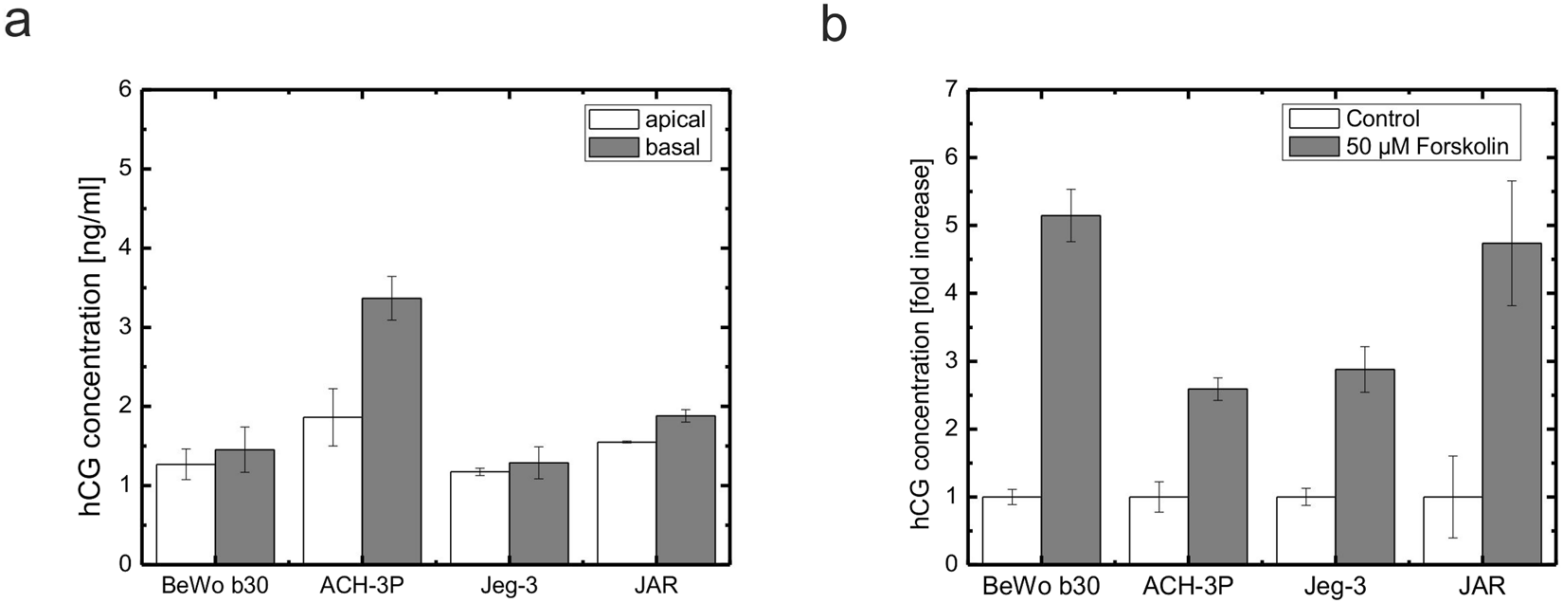
Secretion of hCG of four different placental cell lines. (**a**) Apical and basal secretion of hCG of BeWo, ACH-3P, Jeg-3 and JAR placental transwell cultures at day 5 post seeding (n = 3). (**b**) Response to forskolin treatment of hCG secretion of trophoblast derived cell lines BeWo, ACH-3P, Jeg-3 and JAR placental cell lines in 24-well microtiter plates at day 7 post seeding. Cells were stimulated with 50 µM forskolin at day 5. Data points are mean values ± SD for *n* = *3*. Data is normalized to cell numbers and expressed as fold increase of the untreated controls.

To assess the apical and basal secretion of human chorionic gonadotropin (hCG), cell supernatants of the four trophoblast cell lines from basal and apical transwell compartments were analyzed at day 5 post seeding (Fig. 4a). BeWo, Jeg-3 and JAR cells secreted similar amounts of hCG with apical concentrations of 1.26 ± 0.19 ng/ml, 1.17 ± 0.04 ng/ml and 1.55 ± 0.01 ng/ml, and basal concentrations of 1.45 ± 0.28 ng/ml, 1.28 ± 0.20 ng/ml and 1.87 ± 0.07 ng/ml, respectively. In contrast, ACH-3P cells yielded higher hCG concentrations for both, apical and basal compartment, of 1,86 ± 0.36 ng/ml and 3.36 ± 0.27 ng/ml, respectively.

Furthermore, hCG plays a critical part in fusion of trophoblast cells to form a multinucleated syncytiotrophoblast during placental development as well as placental maturation during pregnancy^55^. To assess differences in upregulation of hCG secretion upon induction with forskolin, which is well known to increase hCG levels (biochemical differentiation) and leads to the formation of multinucleated cells in BeWo cell lines (Supplementary Fig. 2)^56, 57^, the four cell lines were induced with 50 µM forskolin in conventional microtiter plates. After 48 h of induction upregulation of hCG secretion was analyzed at day 7 post seeding (Fig. 4b). hCG levels in cell culture supernatants of BeWo b30 and JAR cells increased with hCG concentrations to 40.09 ± 3.02 ng/ml (5.14-fold increase) and 14.12 ± 2.84 ng/ml (4.73-fold increase), respectively. In contrast, hCG levels of ACH-3P and Jeg-3 cells increased less strong with highest concentrations of 19.65 ± 0.86 ng/ml (2.58-fold increase) and 55.71 ± 6.91 ng/ml (2.87-fold increase), respectively.

### Placental barrier function: nanoparticle transport is highly dependent on the *in vitro* cell model chosen

**Figure 5:**
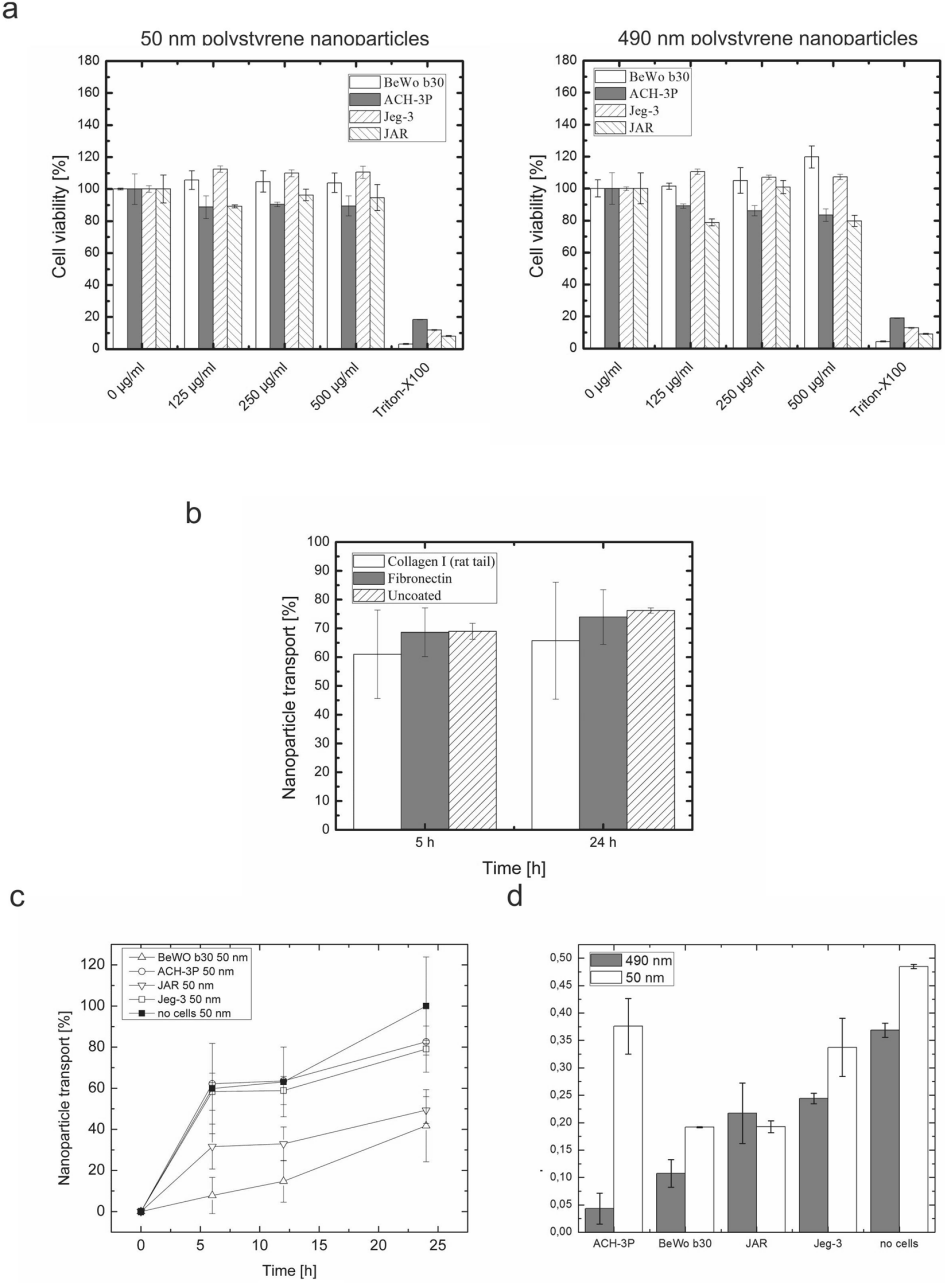
Evaluation of barrier function: Transport of nanoparticles through placental cell barriers. (**a**) Cytotoxicity of fluorescently-labelled 50 nm and 490 nm polystyrene nanoparticles. (**b**) Influence of adhesion promoters on the transport of fluorescently-labelled 50 nm polystyrene nanoparticles through 3 µm transwell membranes. Values for transwell inserts without cells were set to 100% of membrane-less wells. (**c**) Time-trace of transcellular transport after 6 h, 12 h and 24 h of transport of 50 nm fluorescently-labelled polystyrene nanoparticles through BeWo, ACH-3P, Jeg-3 and JAR cell barriers. (**d**) Apical-to-basal transport ratio after 24 h for 50 nm and 490 nm fluorescently-labelled polystyrene nanoparticles through BeWo, ACH-3P, Jeg-3 and JAR cell barriers. Data points are mean values ± SD for *n* = *3*.

To evaluate the size-dependent physiological cut-off as indicator for physiologically relevant placental barrier function, nanoparticle transfer studies in transwell microtiter plate setup was performed for all four placental cell types using 50 nm and 490 nm polystyrene particles (Fig. 5). First, the concentration dependent cytotoxic potential of polystyrene nanoparticles was assessed using an MTT cytotoxicity assay to identify a suitable working nanoparticle concentration (Fig. 5a). Overall, all four cell types incubated with 50 nm as well as 490 nm polystyrene nanoparticles displayed no relevant decrease of viability with values in the range of 85% to 120%. A nanoparticle working concentration of 250 µg/ml was chosen for all subsequent nanoparticle transport studies through placental cell barrier models over a period of 24 h.

Next, the influence of frequently used adhesion promoters on the transfer of nanoparticles through acellular transwell inserts was assessed over a period of 24 h to identify any cell-independent nanoparticle retardation effects (Fig. 5b). Whereas uncoated 3 µm polyester transwell inserts achieved transfer rates of 76.20 ± 0.92% for 50 nm polystyrene particles, membrane surface modifications with fibronectin as well as collagen I yielded slightly reduced nanoparticle transfer to 73.90 ± 9.48% and 65.72 ± 20.31%, respectively. These results indicate that surface modification strategies have an impact on nanoparticle transfer rates most likely caused by increased nanoparticle adhesion and aggregation as well as clogging of the micro-sized pores. Further, standard deviations of transwell inserts with surface modifications were 10 to 20-fold higher compared to pristine surfaces. Therefore, all subsequent nanoparticle transport studies were performed on non-modified transwell inserts.

Finally, the barrier capacity of the four different cell lines was assessed by evaluation of apical-to-basal transport of 50 nm and 490 nm polystyrene beads. First, transport of 50 nm polystyrene nanoparticles was measured to identify the optimal time point for assessment of barrier function of the four cell models (Fig. 5c). After a total period of 24 h a clear difference in transcellular transport was identified as ACH-3P and Jeg-3 were more permeable towards 50 nm sized nanoparticles with 82.58 ± 0.77% and 79.05 ± 11.22% compared to BeWo and JAR cells with 41.74 ± 17.56% and 49.33 ± 6.55%, respectively.

In a next set of experiments, the comparison between transport of 50 nm and 490 nm polystyrene nanoparticles as key characteristic for barrier function was assessed after 24 h of nanoparticle administration at day 5 post-seeding. All barrier cell models showed size-dependent retention of 490 nm polystyrene nanoparticles compared to the acellular control with apical to basal transport ratios of 0.043 ± 0.028, 0.107 ± 0.025, 0.244 ± 0.009 and 0.217 ± 0.055 for ACH-3P, BeWo, Jeg-3 and JAR, respectively. However, Jeg-3 and JAR cells displayed elevated permeability compared to BeWo and ACH-3P. For BeWo and JAR cells similar permeability of 50 nm polystyrene nanoparticles with ratios of 0.191 ± 0.001 and 0.192 ± 0.010 was measured, whereas ACH-3P and Jeg-3 cells showed an increase of basal to apical transport ratio of 0.375 ± 0.050 and 0.337 ± 0.0529 for 50 nm nanoparticles, respectively.

### Placental barrier function does not correlate with differences in metabolic activity or proliferation rate of the cell line tested

To rule out that any of the parameters tested and differences identified is caused rather by differences in metabolic activity or proliferation instead of actual difference in cellular and biologic function, metabolic activity and proliferation assays based on Alamar blue were performed for each of the cell lines. As expected ACH-3P showed highest metabolic activity with fluorescence intensity values of 8144.77 ± 358.50, 15968 ± 2127.05, 40480,33 ± 500,17 for day 1, 3 and 5 post seeding (Supplementary Fig. 2a), respectively. In contrast, JAR, Jeg-3 and BeWo b30 cell lines showed lower values of 31441.88 ± 3033.65, 30811.88 ± 1030.59 and 20135.88 ± 1978.01 at day 5 post seeding, respectively. With respect to population doubling times (Supplementary Fig. 2b) ACH-3P cells showed fastest proliferation with 2.74 ± 0.35 days in contrast to the other three cell lines with lower proliferative capacity of 2.29 ± 0.13 days for BeWo b30, 2.52 ± 0.24 for Jeg-3 cells and 2.40 ± 0.18 days for JAR cells, respectively.

## Discussion

This work shows a comparative study carried out on four frequently used placental *in vitro* cell models. To further validate the currently available options, the aim of this work was to demonstrate how the choice of cell model, even though all of them are considered valid trophoblast models, can affect research outcome gravely. As demonstrated, multiple tiers of physiologically relevant tests are necessary to deduct any evidence on the quality and performance of *in vitro* models for the chosen placental research field (Table 1).

**Table 1:** Functional evaluation matrix for BeWo, ACH-3P, JAR and Jeg-3 cell models.

	Cell line				
	BeWo	ACH-3P	JAR	Jeg-3	Primary model systems
Tight junctions (ZO-1)	+	+	+	+	+^64^
NaFlu retardation	+	+	~	+	+^64^
TEER (day 5)	169.33 ± 3.05	340.44 ± 34.31	179 ± 4.31	308.66 ± 27.95	323.91 ± 35.45^64^
Glucose transport (60 min)	56.9 ± 7.6%	55.2 ± 4.1%	52.0 ± 4.3%	9.1 ± 0.1%	<40%^62^ (term placental explants, 120 min) <5%^64^ (primary trophoblast cells, 60 min)
hCG release (apical/basal) [ng/ml]	1.26/1.45	2.87/5.09	1.6/1.9	1.11/1.22	130.56^64^/−
hCG release (50 µm forskolin stimulus) [fold increase]	5.14	2.58	2.58	4.73	N/A
Polystyrene nanoparticle cut-off (F-to-M ratio of 50 nm/490 nm particle permeability)	0.19 ± 0.001/0.102 ± 0.02	0.37 ± 0.05/0.04 ± 0.02	0.19 ± 0.01/0.21 ± 0.05	0.33 ± 0.05/0.24 ± 0.01	0.4/0.05 (placental perfusion model)^28^

One key parameter of intact placental cell barriers is the establishment of apical-basal polarization which is mainly guided by formation of intercellular tight junctions. Thereby, tight junctions control the paracellular transfer of cells as well as biomolecules, pharmaceuticals, and pathogens^58, 59^. Immunostaining of tight junction proteins such as zona occludens-1 (ZO-1) is considered a standard technique to evaluate quality of lateral cell-cell boundaries. Even though all four cell models showed proper tight junction formation and morphology was evaluated as being proper (continuous borders without high intracellular protein localization), when correlating tight junction morphology with actual functional assays (sodium fluorescein and TEER) barrier integrity over time resulted in major differences between the allegedly tight barriers and similar cell lines. These results indicate that even though the cells may look similar and even have had the same origin at some point of their establishment, in terms of barrier formation as well as expression and localization of tight junction proteins and leakage behavior over a period of 7 days they are not similar at all. This means that depending on the degree of tightness that needs to be achieved, cell type as well as culture duration and initial cell seeding density needs to be considered and chosen carefully. In this study, ACH-3P and Jeg-3 cells showed tight barriers with TEER values similar to human primary trophoblast monolayers with net values around 300–400 Ohms cm^−2^. Also, time-resolved analysis of these complex mechanisms need to be analyzed to enable modelling of different placental *in vitro* scenarios (e.g. tight vs. intermediate vs. leaky).

Trans-placental glucose transport is known to be an important function of the placenta *in vivo* since the fetus has the incapacity to produce enough glucose as energy source. Therefore, facilitated transport through the placental barrier results in glucose transport to and uptake of the fetus^48^. A high glucose uptake and permeability is therefore considered essential for physiological fetal metabolism and growth and is mediated by transporter proteins including glucose transporter isoform-1 (GLUT1) and isoform-3 (GLUT3)^49, 60^. For instance, in pathological pregnancies such as fetal growth restriction and maternal diabetes placental GLUT transporter expression is altered indicating changes in placental glucose transporters occur in abnormal fetal growth^61^. The three cell lines BeWo b30, ACH-3P and JAR displayed high glucose transport, which is more similar to data derived from term placenta explants^62^. In contrast, Jeg-3 cells showed lower transport of glucose, which may be explained by a variation in the activity of GLUT transporters. Previously, a comparative study of Jeg-3 and JAR cells has revealed that Jeg-3 in contrast to JAR cells do not react to hyperglycemia and thus stimulation of the placental glucose transport system^63^, indicating that Jeg-3 cells have a quite different molecular mechanism for transport. Interestingly, glucose transport for Jeg-3 cells does correspond with values from primary trophoblast cell cultures^64^.

Since the placenta is an organ with high endocrine activity, hormone production and release is an important aspect of a placental cell model, especially of a trophoblast cell model. Human chorionic gonadotropin (hCG) is a protein secreted by the placental villous trophoblast from early pregnancy on and its release is a marker of continuous endocrine activity of the trophoblast. It plays a crucial role in placental as well as fetal growth and development including stimulation of the maternal thyroid gland to secrete thyroid hormones, control of transplacental iodide transport via sodium iodide symporters, placental vascularization and syncytial fusion converting the cytotrophoblast to the syncytiotrophoblast^65, 66^. The choriocarcinoma cell lines including BeWo b30, Jeg-3 and JAR showed hCG release in the range of 1.26 to 1.55 ng/ml to the apical side and slightly elevated hCG levels for the basal compartment ranging from 1.28 to 1.87 ng/ml. The human trophoblast cell line ACH-3P, however, displayed a several-fold increased hormone release into both basal as well as apical compartments with values of 1.86 and 3.36 ng/ml for apical and basal compartment, respectively. These elevated levels can be explained due to the higher metabolic activity and proliferation (Supplementary Fig. 2) that stem from ACH-3P being a hybrid cell line established by fusion of first trimester human trophoblast cells with AC1-1 human choriocarcinoma cells^23^. Further, all of the analyzed cell models displayed increased hormone release upon chemical stimulation with forskolin^56, 57^, which is reported to go along with placental cell differentiation and for fusogenic cell lines such as BeWo cell lines comes along with formation of syncytia^47^. Comparing overall hCG levels among the four cell models, Jeg-3 cells produced the highest total amount of hCG, while JAR cells displayed the least sensitivity to react upon forskolin stimulation.

Besides endocrine functions, control of transport is a fundamental function of the placental barrier. Interaction of nanoparticles with cells is known to be size and material dependent^67–69^, due to the formation of a complex protein shell, termed protein corona^70, 71^. Based on nanoparticle transport studies using placental *ex vivo* perfusion models, transcellular transport of polystyrene nanoparticles through the placenta is known to be highly dependent on nanoparticle size. In this study, we used similar polystyrene nanoparticles which have already been demonstrated to have a size-dependent behavior for transport across BeWo placental barrier models with a cut-off around 240 nm^28^. For instance, polystyrene nanoparticles with 50 nm size should penetrate the placental barrier more easily than 100 nm^72^. In this study, ACH-3P and Jeg-3 cell lines displayed higher permeability towards 50 nm polystyrene nanoparticles compared to BeWo b30 and JAR cells at day 7 post seeding. With respect to size-dependent and reduced transport of bigger 490 nm polystyrene nanoparticles, BeWo b30 and ACH-3p cell barrier displayed lower permeability than JAR and Jeg-3 day 7 post seeding. These results indicate that BeWo and JAR cells represent a cellular barrier that 50 nm particles cannot easily pass whereas ACH-3P and Jeg-3 display more physiologically expected behavior with most of the small nanomaterial passing through. However, when comparing the presented results with perfusion data after 3 h derived from *ex vivo* placental perfusion experiments^28^, only ACH-3P cells display similar values of 0.37 ± 0.05 for 50 nm and 0.04 ± 0.02 490 nm polystyrene nanoparticles comparable with *ex vivo* perfusion data. Nonetheless, BeWo b30 showed similar cut-off for 490 nm polystyrene nanoparticles they display reduced transfer for 50 nm polystyrene nanoparticles.

Overall the differences in cellular function presented in this work could be attributed neither to variations in metabolic activity nor proliferation and population doubling rates of the four cell lines. Even though ACH-3P are the fastest proliferating cells with the highest metabolic activity, they displayed similar TEER values as Jeg-3 which are less proliferative and have lower metabolic activity. Further, BeWo b30 cells displaying the lowest population doubling times and metabolic activity showed similar glucose transport rates as ACH-3P. For Jeg-3 cells the reduced glucose transfer rates cannot be explained by differences in proliferation, metabolic activity or cell-to-cell junctions since they displayed similar proliferation rates as BeWo b30 and JAR cells, similar metabolic activity as JAR cells and comparable and high TEER values as ACH-3P.

To conclude, we have demonstrated that *in vitro* cell models need to be carefully considered for functional studies, since selection of cell type can have severe impact on the scientific outcome. For instance, among the tested cell lines the choriocarcinoma cell line BeWo is best suited for studies on syncytial fusion. However, ACH-3P, JAR and Jeg-3 cells react to forskolin treatment with elevated levels of hCG they do not form syncytia^73^ and are therefore poor models for syncytialization over a period of 7 days. Even though all cell lines display proper tight junction formation based on ZO-1 localization, with respect to TEER and electrical behavior ACH-3P and Jeg-3 are more similar to primary cells. Further, there is increasing evidence to suggest that although among the first-trimester trophoblast cell lines BeWo is well established and frequently used for instance for nanoparticle transport studies^22, 24, 56, 57^, the trophoblast cell line ACH-3P can resemble transfer ratios more similar to *ex vivo* placenta perfusion models.

## Materials and Methods

### Cell culture

BeWo b30 cells (kindly provided by Dr. Tina Buerki-Thurnherr, EMPA, Switzerland) were cultivated in Dulbecco’s modified Eagle medium (DMEM with L-glutamine and high glucose; Gibco, 11965-084). ACH-3P cells were cultivated in a 1:1 mix of Dulbecco’s modified Eagle medium (DMEM with L-glutamine and high glucose; Gibco) and Ham’s F12K medium (with L-glutamine; Gibco, 21127-022). JAR cells were maintained in RPMI 1640 medium (with L-glutamine; Gibco, 11875-085). Jeg-3 cells were cultivated in minimum essential medium (MEM with L-glutamine; Gibco, 11095-080). All culture media were supplemented with 10% fetal calf serum (FCS; PAA, A15-101) and 1% antimycotic/antibiotic mix (Gibco, 15240-062). All cells were cultivated in a humidified atmosphere at 37 °C and 5% CO_2_.

### Immunohistochemistry

Visualization of tight junctions (zona occludens 1, ZO-1) of placental cultures on gelatin-coated glass slides and transwell inserts was performed by cell fixation using 2% paraformaldehyde (Sigma Aldrich, 158127) for 20 min at RT, permeabilization with 0.2% Triton X-100 for 10 min at RT and blocking in 5% goat serum (Sigma Aldrich, G9023) for 1 h at RT. A primary monoclonal mouse anti-ZO-1 antibody (Invitrogen, ZO1-1A12) diluted 1:100 in 0.5% HSA/DPBS was incubated over night at 4 °C. Finally, a secondary goat anti-mouse Alexa 488 antibody (Abcam, ab150077) diluted 1:400 in 0.5% HSA/DPBS was incubated for 1 h at RT. Prior to confocal laser scanning microscopy, samples were embedded in Vectashield® mounting medium (Fisher Scientific, NC9265087). Between all steps samples were washed three times in 1x DPBS for 5 min at RT.

For visualization of hCG, BeWo cells were cultivated on chamber slides in medium containing 20 µM forskolin (Sigma, St Louis, MO, USA) for 48 hours to induce syncytialization. After incubation, cells were washed with PBS and air dried. Prior to immunofluorescence staining cells were fixed in acetone for 10 min. After washing with PBS and a blocking step for 7 min, a monoclonal anti ß-hCG antibody (BioLogo, Kronshagen, Germany) was applied for 30 min. Slides were washed with PBS and incubated with Alexa 555 goat-anti-mouse secondary antibody (Invitrogen, Eugene, OR, USA) for 30 min. Nuclei were stained with DAPI (Invitrogen) after washing with PBS and finally slides were mounted with ProLong Gold antifade reagent (Invitrogen). Fluorescence microscopy was performed using an Axiophot microscope with an AxioCam HRc camera (Zeiss, Oberkochen, Germany).

### Confocal laser scanning microscopy

Confocal laser scanning microscopy (CLSM) was performed using a Leica TCS SP5 II system (Leica). Images were recorded with a 63x oil immersion objective using the manufacturer’s LAS AF imaging software.

### Sodium-fluorescein leakage assay

For evaluation of barrier integrity, a sodium fluorescein leakage assay was performed in 12-well transwell microtiter plates (VWR, 734-1580). First, the basal chamber of the transwell inserts was filled up with 1.5 ml of cell culture medium on day 0. Next, 1 * 10^5^ cells cm^−2^ in 0.5 ml culture medium were seeded onto the 3 μm porous polyester membrane of the apical chamber. For no-cell controls, only the respective cell culture medium was pipetted onto the membrane filters. At day 3 post seeding, medium from the apical chamber was aspirated and replaced with 0.5 ml of 5 μM sodium fluorescein in complete culture medium for 1 h. At each time point, 50 μl samples from the basal chamber were pipetted into a 96-well plate and analyzed using an EnSpire 2300 plate reader (Ex. 470/Em. 525; Perkin Elmer). After each measurement, the fluorescein solution was aspirated and cells were maintained in complete culture medium. Results were corrected for loss in sample volume.

### TEER measurements

Placental cells were prepared as described for Sodium-fluorescein leakage assay above. Placental barrier integrity was measured with an EVOM-2 voltohmeter and a STX3 Ag/AgCl electrode (World Precision Instruments Ltd) containing 3 mL of medium on the baso-lateral and 0.5 ml of medium on the apical transwell side. To ensure reproducibility of the measurements and counteract artifacts based on the temperature-dependency of this sensing method, all samples were allowed to cool down for 40 min and data points were measured in full culture medium at a sample temperature of 23 °C without any washing steps. TEER values were corrected for surface area and expressed as Ohms/cm^2^.

### hCG ELISA

For evaluation of hCG secretion, cells were seeded at a density of 1 * 10^5^ cells cm^−2^ in either transwell or 48-well microtiter plates. For hCG upregulation experiments, complete growth medium was supplemented with 50 µM forskolin (Sigma Aldrich, F6886) at day 5 post-seeding. For each time point a sample volume of 100 µl was collected and diluted 1:20 in assay diluent B. The enzyme-linked immunosorbent assay (Abcam, ab100533) was performed as described in the manufacturer’s product protocol. Readout was performed using an EnSpire 2300 plate reader (Abs. 450; Perkin Elmer). All hCG values were normalized to cell numbers.

### Glucose transport assay

For evaluation of glucose transfer through placental cell barriers, cells were seeded at a density of 1 * 10^5^ cells cm^−2^ in 12-well transwell microtiter plates (VWR, 734-1580). First, apical and basal culture medium was replaced with complete DMEM_high glucose_ and DMEM_no glucose_ (Gibco, 11966-025), respectively. For each time point a sample volume of 10 µl was collected and diluted 1:30 in DMEM (no glucose). The glucose assay was performed using a glucose assay kit (Abcam, ab65333) according to the manufacturer’s product protocol. Readout was performed using an EnSpire 2300 plate reader (Ex. 535/Em. 590; Perkin Elmer). The results were corrected for loss of 10 µl sample volume per timepoint.

### Nanoparticle cytotoxicity assay

For evaluation of the cytotoxic potential of non-toxic 50 nm and 490 nm polystyrene nanoparticles (Kisker Biotech, PFP-00552), an MTT (3-(4,5-Dimethylthiazol-2-yl)-2,5-diphenyltetrazolium bromide) assay was performed in 96-well microtiter plates to determine placental cell viability. Cells were seeded at a cell density of 1 * 10^4^ cells per well and propagated for 24 h. As a negative control, no-cells controls were considered. On the first day after the incubation period, cells were exposed to different concentrations of nanoparticles up to a concentration of 500 μg ml^−1^. Subsequently, cells were exposed to nanoparticles for another 24 h. On the second day, cells were washed once with 500 μl pre-warmed PBS, then 100 μl of MTT reagent at a concentration of 5 mg ml^−1^ was applied and plates were incubated for 4 h. For solubilization intracellular formazan crystals, 100 μl of 10% SDS/0.01 M HCl solubilization solution was added and plates were incubated overnight. After solubilization, MTT analysis was performed using an EnSpire 2300 plate reader (Abs. 570 nm).

### Nanoparticle transport assay

For assessment of the influence of frequently used adhesion promotors on trans-placental nanoparticle transport, first, a transport assay of 50 nm polystyrene nanoparticles (PFP-00552, Kisker Biotech) across 3 µm transwell inserts was performed. Transwell membranes were coated with 10 µg/ml fibronectin (F2006, Sigma Aldrich) for 1 h at 37 °C or 2% collagen I from rat tail (C3867-1VL, Sigma Aldrich) overnight at RT. After coating, the transwell inserts were washed three times with PBS to remove excess of adhesion promotors. Next, 1.5 ml of complete culture medium was pipetted into the basal chamber of the transwell inserts. The apical chamber was filled up with 0.5 ml of PS NPs that had a concentration of 500 μg/ml. For each timepoint 100 μl of sample were collected from the basal chamber and analyzed using an EnSpire 2300 plate reader (Ex. 470/Em. 525; Perkin Elmer).

For evaluation of nanoparticle transfer across placental cell barriers, 1 * 10^5^ cells cm^−2^ in 0.5 ml complete growth medium were seeded on apical transwell chambers and incubated. On day 5, medium was replaced in the basal chambers with fresh culture medium and 0.5 ml of 100 μg/ml nanoparticle solution was injected into the apical chamber of transwell inserts. For each time point 100 μl of sample was collected from the basal chamber and analyzed using an EnSpire 2300 plate reader (Ex. 470/Em. 525; Perkin Elmer). The results were corrected for loss of sample volume.

### Data availability

The datasets generated during and analyzed during the current study are available from the corresponding author on reasonable request.

## Electronic supplementary material

Supporting Information
