# Supplementary material for: A comparative study of five physiological key parameters between four different human trophoblast-derived cell lines
Source: Sci Rep. 2017 Jul 19;7:5892. doi: 10.1038/s41598-017-06364-z (PMC5517571; doi:10.1038/s41598-017-06364-z)
Supplement: Supplementary file 1 — Supporting Information [file 41598_2017_6364_MOESM1_ESM.pdf]

Supporting information - A comparative study of five physiological key parameters between four different human trophoblast-derived cell lines

Mario Rothbauer [1,*], Nilaypatel Kumar [3], Hajnalka Gondola[1], Monika Siwetz[2], Berthold Huppertz [2] and Peter Ertl [1]

[1] Vienna University of Technology, Faculty of Technical Chemistry, Institute of Applied Synthetic Chemistry & Institute of Chemical Technologies and Analytics, Getreidemarkt 9, 1060 Vienna, Austria

[2] Medical University of Graz, Institute of Cell Biology, Histology and Embryology, Harrachgasse 21/VII, 8010 Graz, Austria

[3] University of Vienna, Department of Pharmacognosy, Althanstrasse 14, 1090 Vienna, Austria

* mario.rothbauer@tuwien.ac.at

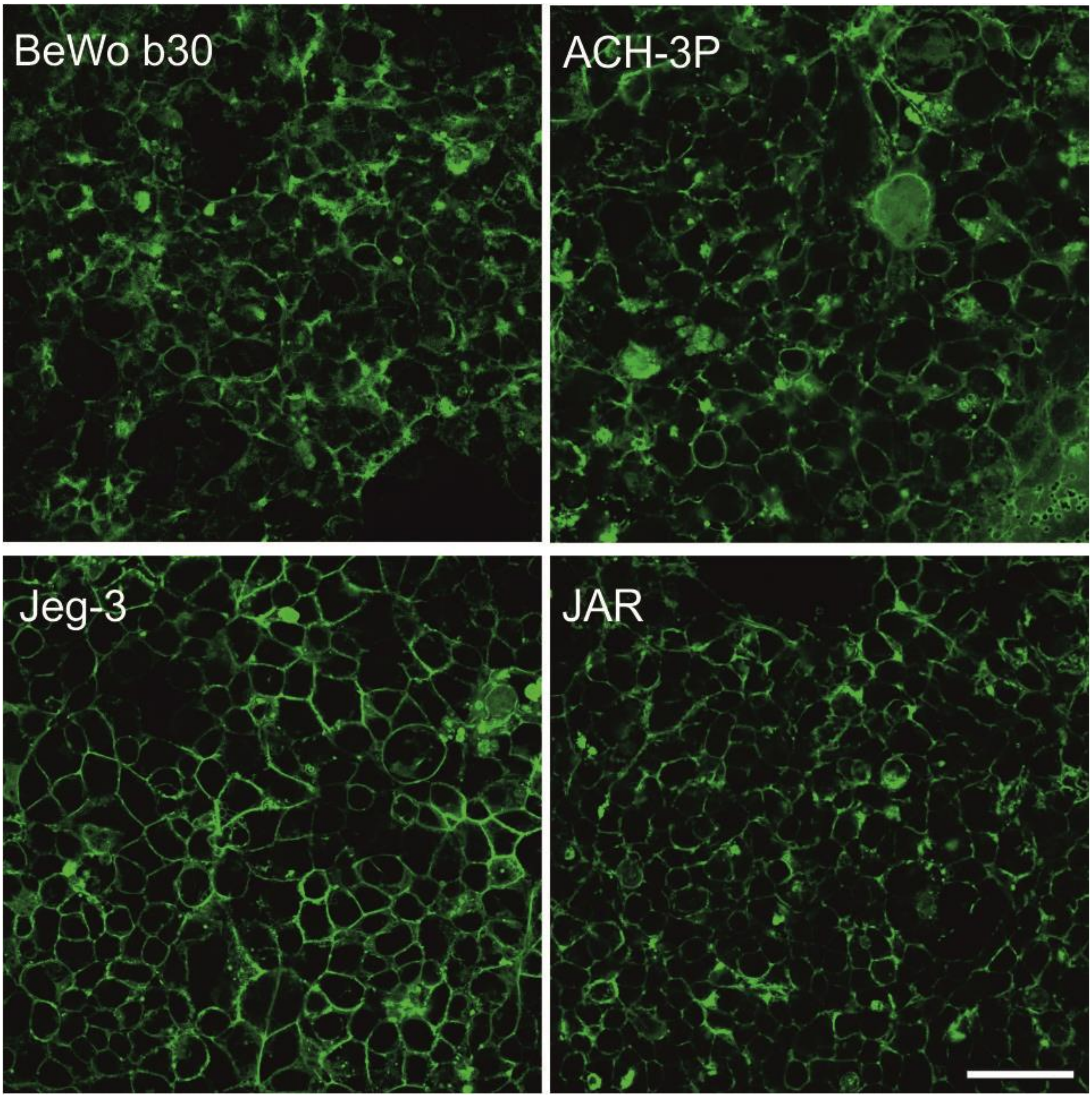

SI Fig. 1 Immunofluorescence stain for ZO-1 of placental cell models grown on 3 µm transwell 3 inserts at day 7 post-seeding.

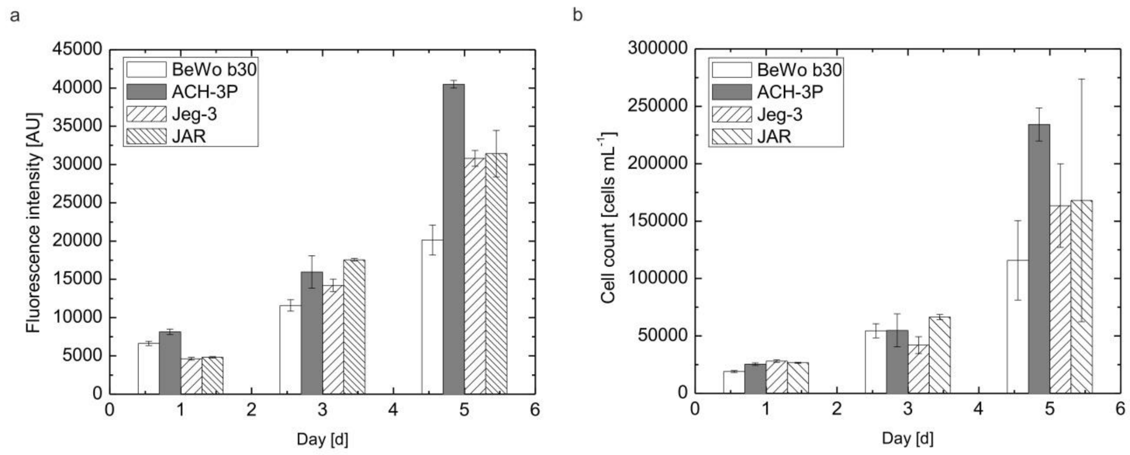

SI Fig. 2 (a) Metabolic activity and (b) proliferation of placental cell models at day 1, 3 and 5 post seeding. Data points are presented as mean values ± SD for n = 3
